# Return of Research Results to Participants in Longitudinal Cohort Studies in Health Research: Findings From 24 Cohorts of the Amsterdam Cohort Hub

**DOI:** 10.1111/hex.70886

**Published:** 2026-10-03

**Authors:** Yuwei Qi, Marieke F. van Wier, Femke Jansen, Marleen van der Horst, Charles Agyemang, Elias Balt, Marieke M. T. Blom, Cyrina Brouwer, Jos A. Bosch, Anouk den Braber, Felix P. Chilunga, Udi Davidovich, Merijn Eikelenboom, Awa A. Gassama, Mirjam I. Geerlings, Janneke Heijne, Melany Horsfall, Martijn Huisman, Kim M. Jonkman, Joep Killestein, Tamira K. Klooker, Anitra D. M. Koopman, Sophia E. Kramer, Jonathan Krikeb, Franca H. Leeuwis, Lannie Ligthart, Saskia Y. M. Mérelle, Ghiselinde B. G. M. Meijs, Tom Molenaar, Mirjam Oosterman, Patricia van Oppen, Nadine P. G. Paans, Brenda W. J. H. Penninx, Didi Rhebergen, Femke Rutters, Yola Sol, Mandy Spaan, Eva M. M. Strijbis, Geertje Marije Takkenberg, Betty M. Tijms, Natasja D. Vijfschagt, Tanja G. M. Vrijkotte, Marlies J. Visser, Eco J. C. de Geus, Cécile R. L. Boot, Emiel O. Hoogendijk

**Affiliations:** ^1^ Department of Epidemiology and Data Science Amsterdam University Medical Center, Location VU University Medical Center Amsterdam the Netherlands; ^2^ Amsterdam Public Health Research Institute Amsterdam the Netherlands; ^3^ Section Ear and Hearing, Department of Otolaryngology‐Head and Neck Surgery Amsterdam University Medical Center, Location Vrije Universiteit Amsterdam Amsterdam the Netherlands; ^4^ Department of Otolaryngology‐Head and Neck Surgery Amsterdam University Medical Center, Vrije Universiteit Amsterdam Amsterdam the Netherlands; ^5^ Cancer Center Amsterdam Research Institute Amsterdam University Medical Center, Vrije Universiteit Amsterdam Amsterdam the Netherlands; ^6^ Department of Public and Occupational Health Amsterdam University Medical Center University of Amsterdam Amsterdam the Netherlands; ^7^ 113 Suicide Prevention, Research Department Amsterdam the Netherlands; ^8^ Department of General Practice Amsterdam University Medical Center, Location Vrije Universiteit Amsterdam Amsterdam the Netherlands; ^9^ Department of Biological Psychology Vrije Universiteit Amsterdam Amsterdam the Netherlands; ^10^ Department of Psychology University of Amsterdam Amsterdam the Netherlands; ^11^ Department of Medical Psychology Amsterdam University Medical Center University of Amsterdam Amsterdam the Netherlands; ^12^ Alzheimer Center Amsterdam, Neurology Vrije Universiteit Amsterdam, Amsterdam UMC Location VU University Medical Center Amsterdam Amsterdam the Netherlands; ^13^ Amsterdam Neuroscience, Neurodegeneration Amsterdam the Netherlands; ^14^ Department of Infectious Diseases, Public Health Service of Amsterdam Amsterdam the Netherlands; ^15^ Department of Social Psychology University of Amsterdam Amsterdam the Netherlands; ^16^ Department of Psychiatry Vrije Universiteit Amsterdam the Netherlands; ^17^ Amsterdam Neuroscience, Mood, Anxiety, Psychosis, Stress, and Sleep Amsterdam the Netherlands; ^18^ Department of Internal Medicine Amsterdam University Medical Center, Location University of Amsterdam Amsterdam the Netherlands; ^19^ Institute for Immunology and Infectious Diseases (AII), Amsterdam University Medical Center Amsterdam the Netherlands; ^20^ Department of Sociology, Faculty of Social Sciences Vrije Universiteit Amsterdam the Netherlands; ^21^ Department of Clinical, Neuro‐ & Developmental Psychology Vrije Universiteit Amsterdam Amsterdam the Netherlands; ^22^ Department of Neurology, MS Center Amsterdam Amsterdam UMC Location Vrije Universiteit Amsterdam Amsterdam the Netherlands; ^23^ Department of Endocrinology and Metabolism & Centre of Expertise on Gender Dysphoria Amsterdam University Medical Center Amsterdam the Netherlands; ^24^ Department of Clinical Child and Family Studies, Faculty of Behavioural and Movement Sciences Vrije Universiteit Amsterdam Amsterdam the Netherlands; ^25^ Department of Psychiatry Amsterdam University Medical Center Location Vrije Universiteit Amsterdam Amsterdam the Netherlands; ^26^ GGZ inGeest Mental Health Care Amsterdam the Netherlands; ^27^ GGZ Centraal, Specialized Mental Health Care Amersfoort the Netherlands; ^28^ Amsterdam Reproduction and Development Research Institute Amsterdam the Netherlands

**Keywords:** cohort study, health research, participants, research results

## Abstract

**Background:**

Returning research results to participants is increasingly recognised as part of participant‐centred health research. How this is implemented in longitudinal cohort studies is not yet well characterised. This study aims to provide a descriptive overview of current result return practices across diverse cohort studies in the Netherlands.

**Methods:**

Drawing on the Amsterdam Cohort Hub, an infrastructure of population‐based and patient‐based cohorts in the Netherlands, representatives of 24 affiliated cohort studies completed a structured questionnaire administered via individual interviews. The questionnaire covered what results are returned, to whom, when and how. Individual‐level and group‐level return were distinguished. Cohorts also reported on motivations for returning results and barriers encountered in doing so. Data were summarised descriptively using frequencies.

**Results:**

Individual‐level results beyond incidental findings were returned by 15 cohorts (65.2%), while group‐level results were returned by 23 cohorts (96%). Individual‐level return primarily involved physical and laboratory measures or was embedded in routine clinical care. Group‐level results were mainly shared through newsletters. Practices varied by cohort type and were shaped by ethical considerations and practical constraints. Concerns about participant misinterpretation and limited resources were the most reported barriers.

**Conclusion:**

Return of research results is widely practised across cohort studies, but varies considerably in approach, shaped by ethical considerations, perceived communication risks, and financial and practical constraints. This descriptive overview of current practices may serve as a starting point for cohorts seeking to reflect on or develop their own result return procedures. Future research should examine the effects of various forms of result return on participant engagement and retention, as well as the role of participant preferences in shaping these practices.

**Patient or Public Contribution:**

This study includes a descriptive inventory of patient and public involvement in longitudinal cohort studies, in the form of result return practices. The findings are directly relevant to research participants, as they concern how cohort studies communicate research results to the people who contribute their data. The results may inform how cohorts develop and improve their participant communication strategies in the future.

## 1 Introduction

The return of research results to study participants in health research has gained increasing attention in recent years, reflecting broader shifts toward open science, transparency, and participant‐centred research [1]. Returning results is often framed as an ethical practice that acknowledges participants' contributions, respects autonomy, and supports trust between participants and researchers [2]. Broadly speaking, returning results corresponds to the ‘inform’ level within public participation frameworks, in which the goal is to provide people with balanced and objective information rather than to involve them in decision‐making [3]. Within patient engagement in research specifically, this represents a foundational level of engagement, distinct from more active forms of partnership or co‐decision making [4]. It may also support collaborative research relationships, potentially contributing to co‐creation approaches in the future [2].

Studies on the return of research results have often addressed individual‐level results, particularly in biomedical domains such as genetics and clinical testing, where results may have implications for participants’ health [5, 6]. Ethical and practical questions in this context include accuracy of findings, their clinical relevance, and the potential consequences of sharing them with participants [7, 8, 9]. While research on result return in clinical and genetic settings has provided important guidance, it has largely been developed outside the context of cohort research. Less is therefore known about how individual‐level results are handled in cohort studies, particularly those collecting a wide range of health data beyond clinical care.

Alongside individual‐level results, there has been growing attention to group‐level return of results. This refers to the sharing of aggregated findings, such as lay summaries, with all cohort participants [10]. These practices are less sensitive than individual feedback but remain important for transparency and trust [11, 12]. They may further support participants' understanding of research outcomes and foster participants to be viewed as partners who help shape research processes and communication rather than passive data providers [13]. Nevertheless, how cohorts implement group‐level return remains inconsistently documented. More broadly, despite growing interest in result return at both the individual and group level, fundamental questions about what should be returned, when and how remain largely unanswered. In a JAMA viewpoint, Wong et al. [14] highlighted that, while returning results is both an ethical obligation and a potential driver of participant engagement, empirical evidence on best practices across different research contexts remains scarce, and called for empirical work to begin charting these practices.

Understanding current practices matters for several reasons. It can inform the development of shared guidelines, support transparency and accountability, and help cohorts improve participant engagement and retention. It may also serve as a baseline for moving toward more participatory research models, in which result return becomes one building block of a broader co‐creative relationship between researchers and participants. Wong et al. [14] argued that fundamental questions about *what* should be returned, *when* and *how* remain largely uncharted in practice. Drawing on the Amsterdam Cohort Hub (ACH), an infrastructure of population‐based and patient‐based cohorts in the Netherlands, this study aimed to provide a descriptive overview of current result return practices across 24 affiliated cohort studies, as well as the motivations for returning results and the barriers cohorts face in doing so.

## 2 Materials and Methods

### 2.1 Study Design

This study used a descriptive cross‐sectional design to map current practices for returning research results to participants in cohort studies that are part of the ACH (www.amsterdamumc.org/cohorthub). ACH is a regional research infrastructure that connects, supports and strengthens cohort studies in health research affiliated with Amsterdam UMC, Vrije Universiteit Amsterdam, and the University of Amsterdam by promoting collaboration, shared expertise, and responsible data practices. The aim is to improve data stewardship and accessibility, and increase the societal relevance and public health impact of longitudinal cohort research.

### 2.2 Participating Cohorts

All cohort studies affiliated with ACH were invited to participate in September 2025, and all 24 currently affiliated cohorts provided information. The participating cohorts cover a broad range of health domains, including mental health, ageing, chronic diseases, reproductive health and lifestyle‐related factors.

Of the 24 included cohorts, 14 were population‐based, and 10 were patient‐based. Population‐based cohorts follow defined populations over time and include studies on early life, development, and parenting (Amsterdam Born Children and their Development cohort, ABCD [15], Generations^2^ [16, 17]), HIV, sexually transmitted infections, and sexual behaviour in men who have sex with men (Amsterdam Cohort Studies, ACS [18]), routine primary care data (General Practice Database – Amsterdam, North Holland, Almere, GPD‐ANHA), urban health and ethnic diversity (Healthy Life in an Urban Setting study, HELIUS [19, 20, 21]), glucose metabolism and diabetes in the general population (Hoorn Studies [22]), autism and other neurodivergent conditions (In Kaart; Netherlands Autism Register, NAR [23]), ageing (Longitudinal Aging Study Amsterdam, LASA [24, 25, 26]), mood disorders in high‐risk families (Mood and Resilience in Offspring, MARIO [27]), aetiology of individual differences in behaviour and health (Netherlands Twin Register, NTR [28, 29, 30, 31]), hearing ability and its psychosocial and health correlates (Netherlands Longitudinal Study on Hearing, NL‐SH [32]), psychosocial autopsy research after suicide (Psychosocial Autopsy Study/113 Suicide Prevention, PSA/113 [33]) and cardiometabolic risk in migrant populations (Research on Obesity and Diabetes among African Migrants, RODAM [34]).

Patient‐based cohorts consist of clinical populations defined by specific conditions or care pathways, including dementia (Amsterdam Dementia Cohort, ADC [35]), transgender health and hormone treatment outcomes (European Network for the Investigation of Gender Incongruence, ENIGI [36, 37]), diabetes care in clinical settings (Hoorn Diabetes Care System cohort, Hoorn DCS [38]), multiple sclerosis (MS Cohort [39, 40]), mental health (Netherlands Study of Depression and Anxiety, NESDA [41, 42]; Netherlands Study of Depression in Older persons, NESDO [43]), head and neck cancer (Netherlands Quality of Life and Biomedical Cohort, NET‐QUBIC [44]), Myalgic Encephalomyelitis/Chronic Fatigue Syndrome (Netherlands ME/CFS Cohort and Biobank, NMCB), Obsessive Compulsive Disorder (Netherlands Obsessive Compulsive Disorder Association study, NOCDA [45]), and fertility treatment exposure and long‐term health outcomes (OvariuMstimulatie En Gynaecologische Aandoeningen, OMEGA [46, 47]).

### 2.3 Data Collection

Information on the return of research results was collected by administering a structured questionnaire to one or more representatives of each cohort. The questionnaire was developed for this study based on Wong et al. [14] (Appendix 1). The questionnaire was sent to each representative in advance, followed by an individual interview to clarify responses. Responses were recorded through written notes taken during the interview and subsequently digitalised. Each interview lasted approximately 30 min. Data collection took place in fall 2025. The number of representatives consulted varied by cohort, as did their roles, which included principal investigators, researchers, field workers, project managers and data managers. The questions covered whether cohorts return results at the individual level and/or group level, the types of results returned, the timing of return, the delivery methods used and the intended recipients. Individual‐level results were defined as information returned to participants about their own measurements, test results, or assessments [9, 48]. Group‐level results were defined as aggregated study findings or summaries communicated to participants through cohort‐organised channels [11]. Group‐level return of results did not include dissemination through scientific publications or presentations at academic conferences. Representatives were also asked whether participant preferences regarding result return have been assessed, and were invited to describe motivations for returning results, perceived barriers and whether procedures are in place to gather feedback from participants about the results they receive. Information on how incidental findings are handled is not included in the descriptive overview of result return practices.

The data collection of all cohort studies participating in the ACH has been approved by authorised medical ethical committees [13, 14, 15, 16, 17, 18, 19, 20, 21, 22, 23, 24, 25, 26, 27, 28, 29, 30, 31, 32, 33, 34, 35, 36, 37, 38, 39, 40, 41, 42, 43, 44, 45, 46]. The current study has been exempted from ethical review since we did not collect any personal data.

### 2.4 Data Analysis

Responses were summarised using frequencies and descriptive comparisons only. Open‐text responses were reviewed and categorised according to three dimensions derived from Wong et al. [14]: *what* results are returned, *when* they are communicated and *how* they are delivered. The dimension of *how* encompasses the delivery method, format, intended recipients and organisational procedures involved in communicating results. To ensure accuracy, all collected information was verified by cohort representatives. Cohorts were classified as either population‐based or patient‐based according to definitions established within the ACH. A patient‐based cohort is defined as a clearly delineated group of patients sharing a specific health condition, treatment or clinical context, systematically followed using care data and additional measurements to generate health information for research or quality improvement. A population‐based cohort is defined as a clearly delineated group of individuals from the general population, selected on non‐disease‐related criteria such as geography, age, or demographics, and systematically followed through repeated or periodic data collection to study changes in health and its determinants. ‘Participants’ refers to individuals enroled in the cohort studies themselves. Where cohorts also involve advisory groups or people with lived experience in shaping their communication practices, this is noted in the tables but distinguished from result return to actual study participants.

## 3 Results

All 24 cohorts provided complete information on their current result return practices (Table 1). Sample sizes ranged from several hundred to more than half a million participants. Only one cohort did not engage in result return yet at the time of data collection. Among the 23 cohorts returning results, all provided group‐level results, while 15 (65.2%) returned individual‐level results. Group‐level results were returned by 14 population‐based and 9 patient‐based cohorts, and individual‐level results by 10 population‐based and 5 patient‐based cohorts. Tables 2 and 3 provide a detailed overview of result return practices, with Table 2 focusing on individual‐level results and Table 3 on group‐level results.

**Table 1:** Overview of participating cohort studies.

Cohort name (abbreviation)	Type	Sample size^a^	Primary focus/health domain	Target population	Year established
1. Amsterdam Born Children and their Development cohort (ABCD)	Population	8266	Early‐life exposures, maternal health, child development	Pregnant women and their children in Amsterdam	2003
2. Amsterdam Cohort Studies (ACS)	Population	3024	Sexually transmitted infections (STIs), HIV infection, related incidence, treatment, pathogenesis, prevention and predictors such as sexual behaviour, psychosocial factors, and drug use	Open cohort of Men who have sex with men	1984
3. Amsterdam Dementia Cohort (ADC)	Patient	7855	Dementia diagnosis and progression	Patients who come to the Alzheimer Center at Amsterdam UMC	2000
4. European Network for the Investigation of Gender Incongruence (ENIGI) endocrinology	Patient	3133	Gender incongruence	Transgender adults	2010
5. Generations^2^	Population	2000	Reproduction, early life, and parenting	Pregnant women and newborns	2009
6. General Practice Database – Amsterdam, North Holland, Almere (GPD‐ANHA)	Population	1.3 million	General practice registry	Patients registered at a GP practice centred around the Amsterdam Metropolitan Area	2003
7. Healthy Life in an Urban Setting study (HELIUS)	Population	24780	Ethnic diversity in health and health care	Adults of Surinamese, Ghanaian, Turkish, Moroccan and Dutch descent in Amsterdam	2011
8. HOORN studies	Population	6301	Glycemic control and development of Type 2 diabetes	Adults aged 40–76 in the Hoorn region	1989
9. HOORN (Diabetes Care System) DCS cohort	Patient	> 15,000 (Open cohort)	Diabetes care	People with type 2 diabetes in primary care from the West‐Friesland region	1998
10. In Kaart	Population	12,673	Neurodivergence/developmental problems (ADHD, dyslexia, autism, etc.)	Individuals with neurodivergence or developmental difficulties and their relatives/families across the Netherlands	2022
11. Longitudinal Aging Study Amsterdam (LASA)	Population	6582	Ageing	Community‐dwelling older adults	1991
12. Mood and Resilience in Offspring (MARIO)	Population	593	Intergenerational transmission of depression and anxiety	Children of parents with psychiatric problems	2019
13. MS Cohort	Patient	8138	Multiple Sclerosis (MS)	People receiving MS care	2000
14. Netherlands Autism Register (NAR)	Population	4000	Autism, development, genetics, wellbeing	Individuals with autism	2013
15. Netherlands Study of Depression and Anxiety (NESDA)	Patient‐control	3348	Depression and anxiety	Adults aged 18–65 at inclusion	2004
16. Netherlands Study of Depression in Older persons (NESDO)	Patient‐control	510	Late‐life depression	Adults aged 60+ at inclusion	2007
17. Netherlands Quality of life and Biomedical Cohort (NET‐QUBIC)	Patient	1001	Head and neck cancer	Newly diagnosed patients and informal caregivers	2013
18. Netherlands Longitudinal Study on Hearing (NL‐SH)	Population	3258	Hearing loss, hearing rehabilitation	Adults aged 18–70 at inclusion	2006
19. Netherlands ME/CFS Cohort and Biobank (NMCB)	Patient	2160	Myalgic Encephalomyelitis/Chronic Fatigue Syndrome (ME/CFS)	Patients with ME/CFS	2024
20. Netherlands Obsessive Compulsive Disorder Association study (NOCDA)	Patient	419	OCD course and treatment outcomes	Adults with OCD	2005
21. Netherlands Twin Register (NTR)	Population	280,569	Aetiology of individual differences in behaviour and health	Twins and their parents, siblings, spouses and children	1986
22. OvariuMstimulatie En Gynecologische Aandoeningen (OMEGA)	Patient	40,613	Fertility treatment exposure and long‐term health of women and children	Women who received ovarian stimulation for assisted reproductive technology (*n* = 30,625) and subfertile women not treated with assisted reproductive technology (*n* = 9988) Children conceived by assisted reproductive technology (*n* = 51,417) and children born to parents with subfertility problems without assisted reproductive technology (*n* = 37,832)	1995
23. Psychosocial autopsy study/113 Suicide Prevention (PSA/113)	Population	1563	Suicide prevention strategies	Bereaved family members and friends after suicide	2020
24. Research on Obesity and Diabetes among African Migrants (RODAM)	Population	5114	Diabetes and obesity in African migrants	Adults of Ghanaian origin	2012

^a^ Sample sizes reflect the most recently available data at the time of data collection (2025). Cohorts with ongoing participant inclusion may report higher numbers at a later date.

**Table 2:** Return practices by cohort, individual‐level results (what, when and how).

	What	When	How		
Cohort (Abbreviation)	Individual results	Timing	Recipients	Methods	Organisation
1. Amsterdam Born Children and their Development cohort (ABCD)	Metabolic profile and, in some sub‐studies, accelerometer‐based physical activity summaries.	Timing differs across phases; results are provided either immediately or after laboratory processing.	Participants (Mothers during pregnancy and childhood. Results shift to the adolescent participant once they turn 16.)	Personalised letters by post or results handed directly during visits, depending on the study phase	An independent medical doctor, as specified in the study protocol, evaluates which results are appropriate and interesting to give back.
2. Amsterdam Cohort Studies (ACS)	Laboratory results for HIV and four other sexually transmitted infections (Neisseria gonorrhoeae, Chlamydia trachomatis Treponema pallidum (syphilis) and Hepatitis C virus	As soon as possible after each clinical visit.	Participants. Results may be shared with their general practitioners (GPs) only when the participant explicitly requests this.	Secured digital platform supported by SMS or phone calls for communication and follow‐up.	Results are first processed by the laboratory staff, reviewed by a medical doctor and released to the portal. Participants receive an email or SMS that they can access their results through a secured digital portal. Negative test results are communicated through the portal, as well as positive bacterial test results; the latter is followed by a request and link to receive treatment. Positive HIV test results are not communicated through the portal but face to face initiated by a phone call.
3. Amsterdam Dementia Cohort (ADC)	Return of individual results is embedded in routine clinical care. Clinical and research assessments are integrated within the diagnostic trajectory. Patients automatically receive clinical results via the standard patient portal, and results are discussed during consultations with the neurologist. For research sub‐studies involving non‐patient participants, individual results are not routinely returned but may be provided upon request.	During or shortly after the clinical visit. For research‐only participants who request access, timing depends on availability of a medical doctor.	Patients receive their own results as part of clinical care. GPs are notified when clinically relevant findings are identified. Family members may receive information when a patient lacks capacity.	Patient portal, email or personalised letter by post, phone calls and in‐person meetings during routine clinical consultations.	Neurologists and medical doctors determine and communicate individual results. The research team supports communication for non‐patient research participants.
4. European Network for the Investigation of Gender Incongruence (ENIGI) endocrinology	In ENIGI, data are collected as part of standard clinical care but analysed anonymously at the network level, which means individual participants cannot be identified and individual‐level results cannot be returned.	Not applicable.	Not applicable.	Not applicable.	Not applicable.
5. Generations^2^	Generations^2^ does not return individual‐level research results to participants.	Not applicable.	Not applicable.	Not applicable.	Not applicable.
6. General Practice Database ‐ Amsterdam, North Holland, Almere (GPD‐ANHA)	All data in the GPD‐ANHA are pseudonymized, which makes it impossible to trace information back to specific patients. Because individual participants cannot be identified, no individual findings can be communicated. For research with patient or GP involvement via the network, the researcher is responsible if they return individual‐level results.	Not applicable.	Not applicable.	Not applicable.	Not applicable.
7. Healthy Life in an Urban Setting study (HELIUS)	Laboratory results based on blood and urine samples, as well as anthropometric and physiological measurements. The result letter is available in Dutch, Turkish and English.	Within approximately 2–3 weeks after the participant's visit, following a fixed delivery schedule.	Participants and their GPs.	Personalised letters by post or email (participants may choose their preferred format).	The management team and daily board prepare the content. Results are checked for values above the agreed cut‐off thresholds (in that case the GP is called about the results). The study coordinator oversees organisation and delivery.
8. HOORN Studies	Laboratory results based on blood and urine samples, as well as anthropometric and physiological measurements. Questionnaire‐based results are not returned because they are considered difficult to interpret outside a clinical context, although participants may request those results and get them.	As soon as possible after data collection. Approximately 90% of participants receive their results within 1 month.	Participants and their GPs, the latter only when there are clinically relevant abnormal values.	Personalised letters by post	Results are checked first by the research nurse. Medical doctor from the HOORN Studies reviews the results to identify clinically relevant abnormalities.
9. HOORN (Diabetes Care System) DCS cohort	Routine care data (For subgroup research measurements (e.g. imaging, questionnaires), practices follow those of the Hoorn Studies)	During clinical visits.	GPs	In‐person meetings	Routine care data are returned to GPs and discussed during regular consultations. For additional research measurements conducted in subgroups, such as imaging or questionnaires, result return follows the same practices as the Hoorn Studies.
10. In Kaart	Questionnaire feedback. All participants receive a standardised set of results. The approach is closely aligned with the practice used in the Netherlands Autism Register (NAR), where participants also receive structured feedback derived from annual questionnaires. Because In Kaart includes multiple neurodivergent groups and affiliated organisations, the content is tailored to remain broadly relevant across different subgroups.	Once per year, after completion of the annual assessment.	Participants	Secured digital platform or email.	Content is drafted by the steering group and working group. The project manager prepares the material and submits it for approval. The data manager coordinates delivery.
11. Longitudinal Aging Study Amsterdam (LASA)	Laboratory results based on blood samples and physiological measurements.	As soon as possible after data collection.	Blood test results are sent to the participants' GPs after the examination. Participants may contact their GP to receive their results and any further recommendations	Personalised letters by post (which are sent to the GPs.	The principal investigator and study coordinator are involved in determining content. For blood work, the biobank coordinator is responsible for managing the return process.
12. Mood and Resilience in Offspring (MARIO)	Individual findings are provided only when participants request them.	Upon participant request.	Participants who request their own results.	Direct communication as needed.	Field workers and project coordinators
13. MS Cohort	All individual‐level information, including MRI findings, biomarkers and questionnaire outcomes, is returned to patients through their treating neurologist as part of routine clinical care. These results inform conversations during consultations and influence treatment decisions, such as medication adjustments or monitoring plans. Individual return is therefore considered part of standard patient management rather than a cohort activity.	During clinical visit or when new information becomes relevant for treatment planning. There is no research‐specific timeline.	Participants receive their own clinical results directly from their treating physician. No results are returned to GPs or family members through the cohort structure.	In‐person clinical consultations and phone calls initiated by clinicians.	Clinicians involved in patient care (neurologists or treating physicians) return and discuss individual results. This is not handled by the cohort team.
14. Netherlands Autism Register (NAR)	Personalised online dashboard. Each participant receives a unique link that provides access to their own questionnaire scores, the ability to compare their current scores with earlier years and an overview of how their results relate to the broader NAR population. The dashboard provides wellbeing‐related information only and does not offer clinical diagnoses or medical advice.	As soon as possible after data collection, typically within approximately 6 months.	Participants	Secured digital platform portal with individual score and personalised feedback. Additional contact by email or phone only in urgent situations.	Content is prepared by the principal investigator, study coordinator and researchers. Feedback is given by a participant panel. Delivery is managed by the dedicated communication team.
15. Netherlands Study of Depression and Anxiety (NESDA)	Laboratory results based on blood samples, as well as anthropometric and physiological measurements. Questionnaire‐based results are not returned because they are difficult to interpret without clinical context, but participants may request them.	After each data collection round, once the lab results have been processed. There is no fixed timeline for questionnaire results because they are returned only on request.	Participants and their GPs. Participants requesting questionnaire results receive them directly.	Personalised letters by post, email, and phone calls.	The study receives results from the laboratory, prepares them in a readable format and sends them to participants and their GPs. Research assistants send the results.
16. Netherlands Study of Depression in Older persons (NESDO)	Written summary of their interview so they can check whether the content accurately reflects what they shared. This form of return is used for data validation and is considered part of the research procedure.	As soon as the summary is completed, typically within 1 month after the interview.	Participants	Personalised letter by post, email or telephone, depending on the participant's preference expressed at the end of the interview.	The researcher executing the study assisted by a junior researcher that were responsible for the content. Delivery is carried out by a research assistant or junior researcher.
17. Netherlands Quality of life and Biomedical Cohort (NET‐QUBIC)	Laboratory results based on blood samples, as well as anthropometric and physiological measurements. Some functional test results collected during home visits, such as grip strength, are communicated directly to participants. Questionnaire results are not returned because they are considered difficult to interpret without clinical context.	As soon as possible after data collection, typically within 3 months.	Participants and their GPs.	Personalised letters sent by post, phone calls and in‐person communication during home visits.	Laboratory results are reviewed by a medical doctor; research assistants support delivery.
18. Netherlands Longitudinal Study on Hearing (NL‐SH)	Hearing test that participants complete during the study. Participants receive information about whether their hearing performance is within the normal range. If the test suggests reduced hearing or if participants express concerns, they are advised to seek professional clinical evaluation.	Immediately after the test is completed.	Participants	Immediate on‐screen feedback during the hearing test, with verbal or written advice to seek further evaluation when appropriate.	The principal investigator and the study coordinator.
19. Netherlands ME/CFS Cohort and Biobank (NMCB)	NMCB does not routinely return individual‐level research results.	Not applicable.	Not applicable.	Not applicable.	Not applicable.
20. Netherlands Obsessive Compulsive Disorder Association study (NOCDA)	NOCDA does not return individual‐level research results to participants.	Not applicable.	Not applicable.	Not applicable.	Not applicable.
21. Netherlands Twin Register (NTR)	The twin's zygosity (monozygotic/dizygotic) is returned (unless participants indicated not to want to know). Questionnaire‐based results are not returned because they are considered difficult to interpret outside a clinical context, although participants may request to see the answers they provided. For experimental sub‐studies, personalised feedback is provided on specific measures such as fitness test results, smartphone‐based behavioural data, weekly wellbeing assessments and social media use. No clinical interpretation or advice is given.	As soon as possible once laboratory/experimental results or digital data processing are complete.	Participants	Email, secured digital platform. NTR takes extra care that email addresses belong to the intended individual and not to the whole family or the co‐twin.	Content development involves the entire department. Interpretation of DNA‐based zygosity results is the responsibility of the genotype data manager. Organisational responsibilities are with the project team, and these differ by project but are always supervised by an NTR senior.
22. OvariuMstimulatie En Gynecologische Aandoeningen (OMEGA)	OMEGA does not return individual‐level results. The cohort is retrospective and based on linking historical fertility treatment records with health outcomes, which means that both exposures and outcomes occurred in the past. Because participants are not clinically examined within the study, individual findings or incidental results do not arise. Therefore, returning individual results is not applicable.	Not applicable.	Not applicable.	Not applicable.	Not applicable.
23. Psychosocial autopsy study/113 Suicide Prevention (PSA/113)	PSA/113 does not return individual‐level research results to participants.	Not applicable.	Not applicable.	Not applicable.	Not applicable.
24. Research on Obesity and Diabetes among African Migrants (RODAM)	Laboratory results based on blood samples, as well as anthropometric and physiological measurements.	Within approximately 6 months after data collection.	Participants and their GPs.	Personalised letter by post and phone calls.	Results are first sent to the GP, who is responsible for interpreting the findings and communicating clinically relevant information to the participant.

**Table 3:** Return practices by cohort, group‐level results (what, when and how).

	What	When	How		
Cohort (Abbreviation)	Group results	Timing	Recipients	Methods	Organisation
1. Amsterdam Born Children and their Development cohort (ABCD)	Summaries of overall study progress, key results and updates on ongoing research projects; these include lay‐language factsheets, social media posts and broader public engagement activities such as a co‐creation festival.	Once per year, plus an annual overview.	Participants (Mothers during pregnancy and childhood. Results shift to the adolescent participant once they turn 16)	Newsletter, study website, LinkedIn and Instagram, in‐person co‐creation activities.	The principal investigator, project coordinator and research assistants select, prepare and deliver group‐level communications.
2. Amsterdam Cohort Studies (ACS)	Highlights of ongoing scientific studies. In years when a participant gathering is organised, this event replaces the newsletter and serves additionally as a social meeting in addition to providing a scientific update.	Once per year. In any given year, participants receive either the annual newsletter or the in‐person gathering, not both.	Participants	Newsletter and in‐person meetings.	Study nurses, researchers and the principal investigator prepare the newsletter content. Delivery is organised by study nurses.
3. Amsterdam Dementia Cohort (ADC)	Group‐level findings through annual research update events for professionals, Lunch and Learn sessions for the general public, dementia‐focused outreach events such as Alzheimer op Wielen and regular updates on the ADC website and social media. Newsletters provide additional lay summaries of ongoing work.	Research update events occur once per year. Lunch and Learn meetings are held monthly. Other outreach activities and online updates occur throughout the year without a fixed schedule.	Patients, family members, health care professionals, and stakeholders interested in dementia research.	Newsletter, in‐person meetings, webinars, blogs, social media posts and study website updates.	Content is developed by the principal investigator, researchers and the communication team. Organisation and delivery of events and online communication are managed by the communication and event teams.
4. European Network for the Investigation of Gender Incongruence (ENIGI) endocrinology	No structured return of aggregate or group‐level results to participants is reported. However, study findings are presented at scientific and clinical meetings where patients and practitioners are welcome to attend. These sessions are open to the general patient community but are not specifically directed at ENIGI participants.	Not applicable.	Not applicable.	Not applicable.	Not applicable.
5. Generations^2^	Newsletter that provides an update on study progress and key findings.	Once per year	Participants: in the case of children, results are addressed to mothers.	Newsletter	Content is prepared collaboratively by researchers and the principal investigators, sometimes together with master's students depending on the research project. Delivery and organisation are managed by the project manager.
6. General Practice Database – Amsterdam, North Holland, Almere (GPD‐ANHA)	Summaries of study progress, research outputs and network developments.	4–5 newsletters per year and once per year during the network event.	General practitioners (GPs) in the network and other stakeholders.	Newsletter, annual network meeting, and digital communication channels used within the GP network.	Content is prepared collaboratively by researchers and coordinators. Delivery and logistics are handled by dedicated communication staff.
7. Healthy Life in an Urban Setting study (HELIUS)	Group‐level findings, summaries of scientific publications and infographics that translate study results into lay language.	No fixed timeline. Group‐level updates are shared periodically when new publications or communication materials are available.	Participants	Newsletter, webinars.	Content is prepared and delivered by the study coordinator and scientific coordinator.
8. HOORN Studies	Selected results based on specific research projects and on what is considered relevant or interesting for participants. Not all publications are communicated. Dissemination also includes outreach to the general public through podcasts and other media.	No fixed timeline.	Participants	Newsletter, letter by post, email, podcasts and in‐person meetings.	Communication is handled by the principal investigators and the steering team. Organisational responsibilities vary by project.
9. HOORN (Diabetes Care System) DCS cohort	HOORN DCS employs the same returning practices as the HOORN Studies for group‐level results.				
10. In Kaart	Summarise trends across the cohort and show how individual response patterns compare with group averages. This policy is similar to the Netherlands Autism Register (NAR), where yearly aggregate findings are shared with participating communities.	Annually in the fall.	Participants and organisations representing neurodivergent groups involved in the cohort.	Newsletters and email.	The steering group and working group approve the content. The data manager manages organisation and distribution.
11. Longitudinal Aging Study Amsterdam (LASA)	Besides its newsletter, LASA periodically organises in‐person events under the name LASA on Tour. During these events, researchers present recent findings, provide updates on ongoing projects and engage directly with participants. The events combine scientific presentations and opportunities for informal interaction.	The newsletter is sent once per year. LASA on Tour events are scheduled irregularly; for example, the first such gathering under this name was held on 26 April 2024.	Participants: LASA on Tour events are open to current participants	Printed or digital newsletters, plus in‐person participant meetings (LASA on Tour), including presentations, posters, and direct engagement with the research team.	The LASA research team, including the scientific director, fieldwork coordinators and interviewers, coordinates content development and event organisation.
12. Mood and Resilience in Offspring (MARIO)	Group‐level results are planned but have not yet been implemented because the study is still in the data‐cleaning phase in 2025. Current newsletters focus on general study updates, such as recruitment progress and staffing, rather than research findings. The MARIO website is also used for general communication.	Not applicable.	Not applicable.	Not applicable.	MARIO is a multi‐centre project. Content will be curated collaboratively by field workers, project coordinators and researchers at all participating sites. Each centre has one designated person responsible for gathering and providing input. The steering committee approves the final content. The data manager prepares the mailing list, and research assistants send the communication materials.
13. MS Cohort	Study findings, new projects and ongoing research activities. These updates focus on scientific progress rather than clinical care.	No fixed schedule. Group‐level information is shared when relevant updates are available.	Participants	Newsletter, social media updates, website communication and an annual open day.	The communication team is responsible for preparing content and organising delivery of group‐level results.
14. Netherlands Autism Register (NAR)	Yearly cohort‐wide updates, aggregate patterns in wellbeing, functioning and social participation. Besides, NAR organises around 2‐3 webinars per year, in which researchers present their outcomes to an online audience that can also ask questions.	Newsletter was sent out every 3 months.	Participants, municipalities and the wider public who access NAR materials online.	Newsletter, website updates (https://cijfers.autismeregister.nl/), social media communication and written summaries for municipalities.	A dedicated communication team is responsible for preparing content, organising mailing lists and delivering updates.
15. Netherlands Study of Depression and Anxiety (NESDA)	Lay‐language summaries written for the newsletter and accessible updates on the NESDA website. As of 2025, NESDA will switch from extensive printed newsletters to shorter online newsletters sent at least twice per year. There is also a NESDA Day, although this event is mainly for researchers and study personnel rather than for participants.	Online newsletters are sent at least twice per year. Website updates and publication summaries follow the availability of new research outputs.	Participants	Newsletter, website updates and printed newsletters in earlier years.	Research assistants and field workers propose topics, principal investigators and researchers contribute content and data managers curate mailing lists and manage delivery.
16. Netherlands Study of Depression in Older persons (NESDO)	Updates on study results once analyses are completed. There have also been several NESDO Days, although this event is mainly for researchers and study personnel rather than for participants.	Newsletters are distributed approximately once per year, although there is no fixed schedule. Group results are shared only when analyses are complete and ready to be communicated.	Participants	Newsletter, website updates.	The principal investigator prepares the content. Organisation and delivery are handled by a research assistant or junior researcher.
17. Netherlands Quality of life and Biomedical Cohort (NET‐QUBIC)	Updates on study progress and summarises key findings. The study website is intended mainly for researchers rather than for participant communication (https://www.kubusproject.nl/).	There is no fixed timeline.	Participants	Newsletter delivered by post.	Principal investigators and researchers prepare the content. Newsletter delivery is organised by research assistants.
18. Netherlands Longitudinal Study on Hearing (NL‐SH)	Lay‐language summaries of recent publications, project‐specific updates and general information about changes within the cohort. Summaries are written to be understandable for a wide age range, reflecting the cohort's participant profile (mostly individuals aged 50 to 80, with varying degrees of hearing loss and use of hearing aids).	Roughly three times per year. There is no fixed timeline.	Participants	Newsletter distributed by email or post, depending on participant contact information. The study has also surveyed participants about preferred formats for receiving results. Most preferred newsletters, but a smaller subset expressed interest in videos, webinars, physical meetings, podcasts, email updates or sign‐language supported materials.	The principal investigator and study coordinator are responsible for both content development and the organisation of delivery.
19. Netherlands ME/CFS Cohort and Biobank (NMCB)	Lay summaries of published papers, infographics and statistical summaries created for participants and the wider public. Results from each of the 13 consortium projects are also shared with patient representatives who are connected to those specific projects.	There is no fixed timeline. As soon as findings become available, they are posted to the website and disseminated through the listed channels. Biweekly social media posts (facebook, Instagram) provide brief vlogs. Every 6 months, a newsletter informs all enlisted participants about new findings and developments. Yearly, a general assembly meeting provides recorded research lectures made available to patients and general public through social media and website.	Participants, patient organisations, researchers, funding bodies, general public.	Newsletter, social media updates, infographics, website materials, conference presentations and meetings with stakeholders are posted on social media. Participants can opt out of receiving the newsletter but cannot opt out of publicly shared group results.	A communication working group, which includes patient representatives from relevant patient organisations, carers, researchers and research team members, is responsible for organisation and delivery. Group‐level content is prepared by the principal investigators or project managers of each of the 13 research projects. The communications working group is led by the NMCB project manager.
20. Netherlands Obsessive Compulsive Disorder Association study (NOCDA)	Summarises of study progress and selected findings in lay language.	Once per year, depending on available staff capacity.	Participants.	Newsletter delivered by mail or email.	The principal investigator is responsible for content. The study coordinator and data manager organise and deliver the newsletter.
21. Netherlands Twin Register (NTR)	TwinInfo newsletter (https://tweelingenregister.vu.nl/nl/twinfo‐ons‐digitale‐magazine) consisting of short, accessible summaries of research findings written specifically for participants and their families. Participant portal (mijnntr.nl), where summaries and results of research projects are shared with participants who were part of the projects. Collaboration with the NVOM (Dutch society for parents of multiples): participation in the yearly NVOM meeting day for twin parents, writing articles for the NVOM magazine. NTR also occasionally organises a public‐facing twin event in, e.g., a theme park or zoo, aimed at engaging twins and promoting interest in the research, although this is considered more of a public relations activity than a formal result‐return mechanism.	The Twinfo appears once per year. Timing of feedback on project‐specific results may vary depending on project progress and available staff capacity.	Participants and stakeholders (e.g. funders, faculty boards, advisory boards, teachers reporting on young twins, society for parents of multiples). Website and social media target the general public.	Newsletter, website, social media (LinkedIn, Insta), in‐person meetings (twin events, project‐specific information events).	General content is generated collaboratively within the department, coordinated by the Communication team. Organisation and delivery of project‐specific information responsibilities lay with the project team, and these differ by project but are supervised by an NTR senior.
22. OvariuMstimulatie En Gynecologische Aandoeningen (OMEGA)	Summaries of study progress, response rates and key epidemiological findings, such as associations and cumulative incidences of cancer after fertility treatments. These findings are shared through newsletters, lay‐language summaries of publications, statistical updates and infographics distributed via the study website and through patient organisations.	Group‐level results are most frequently returned during the cohort set‐up and recruitment phase, when updates on invitations and participation rates were communicated. Results of completed studies, such as cancer risks after fertility treatment, are shared once results are published in a scientific journal	Participants and patient organisations.	Newsletter, email, study website and on the websites of patient organisations.	Content is prepared by the researcher or study coordinator together with the principal investigator(s). Organisation and delivery of communication are handled by the study coordinator.
23. Psychosocial autopsy study/113 Suicide Prevention (PSA/113)	Report accompanied by factsheets that summarise key findings in accessible language. Earlier in the study, participants were consulted for feedback on quotations and interpretations, but this became infeasible as the study expanded. Group‐level results are also shared on the 113 website, through traditional media (television, radio, newspapers) and social media channels, and webinars are organised. These webinars are public‐facing rather than specifically designed for study participants. The study has a responsibility to disseminate findings to inform suicide prevention policy, and results are therefore shared broadly with relevant stakeholders.	Once per year through the annual report. Additional materials on the website and public webinars occur when new findings are available. There is no fixed schedule beyond the annual report.	Participants: Results are also disseminated to sectors involved in suicide prevention and policymakers, because 113 has an obligation to share findings that contribute to societal impact.	Report, email, website, webinars and WhatsApp communication. WhatsApp is noted to have very short response times.	Content is prepared by the study coordinator and principal investigator, assisted by communication advisor. The study coordinator is responsible for organisation and delivery.
24. Research on Obesity and Diabetes among African Migrants (RODAM)	Community‐based presentations summarising key findings such as prevalence of high blood pressure and other outcomes. Lay summaries and general findings are also shared after data analyses are completed.	After each data analysis cycle and through ongoing community outreach activities held four times per year.	Participants and community members who attend outreach meetings.	In‐person community meetings, letters delivered by post and other outreach communication channels used during community engagement sessions.	The study coordinator and principal investigator prepare content and organise delivery of group‐level results.

### 3.1 What Is Returned

Among cohorts that return individual‐level results, information shared with participants was largely restricted to measures that are considered straightforward to interpret without extensive clinical guidance, such as laboratory values and basic clinical indicators including blood pressure, body mass index and glucose levels (Table 2). This selective approach was most evident in population‐based cohorts. Most cohorts did not return complex outcomes such as cognitive scores, mental health assessments or questionnaire‐based results. Only a small number of cohorts, notably In Kaart and NAR, provided selected questionnaire‐based feedback, and some cohorts allowed individual access on request. In patient cohorts, individual‐level return mainly consisted of clinically relevant findings generated as part of routine diagnostic or monitoring procedures, rather than results specific to the research activities of the cohort.

Group‐level results were returned by nearly all cohorts and showed substantial consistency in content across both population‐based and patient cohorts (Table 3). Group‐level return commonly consisted of summaries of study progress, key findings from scientific research, and updates on ongoing or planned research activities. In addition to reporting scientific study results, several cohorts also included information on recruitment status, follow‐up waves or changes in study procedures. Group‐level results were generally translated into lay language and shared in accessible formats.

### 3.2 When Results Are Returned

Three broad timing patterns were observed across cohorts (Tables 2 and 3). For individual‐level results, the most common approach was to communicate results as soon as they were available after data collection and processing. In patient cohorts, timing was aligned with clinical procedures, with results communicated during consultations or standard care encounters. In a smaller number of cohorts, individual‐level results were provided only upon participant request. Group‐level result return was more closely linked to communication activities (Table 3). Newsletters were typically distributed at least once per year. Other group‐level activities, such as in‐person meetings or public events, also generally occurred on a yearly basis or in connection with specific study milestones. In addition, some cohorts shared group‐level updates more flexibly, with timing determined by the availability of new findings or completed analyses.

### 3.3 How Results Are Returned

In terms of recipients, individual‐level results were primarily returned directly to participants. In many cohorts, particularly those returning laboratory or clinical measurements, individual results were also shared with their general practitioners (GPs). In cohorts involving children, recipients could change over time as participants aged. For example, in ABCD, results were initially communicated to mothers during pregnancy and early childhood, with responsibility gradually shifting to the child once they reached the age of 16. In patient cohorts, individual‐level results were communicated to participants as part of clinical care, typically by the treating clinician, with GPs informed when this was part of standard care pathways.

Group‐level results were directed to participants collectively, using cohort‐wide communication channels. In addition, several cohorts reported sharing group‐level findings with broader audiences, including healthcare professionals, patient organisations, community groups, or other stakeholders, particularly in studies with a strong public health or societal focus. For example, PSA/113 reported that group‐level findings are disseminated beyond participants to inform suicide prevention policy, including communication with relevant sectors and policymakers such as the Dutch Ministry of Health, Welfare and Sport.

Figure 1 summarises the methods used by cohorts to return individual‐level and group‐level research results. Bars represent the number of cohorts using each method. Individual cohorts could report using more than one delivery method; categories are therefore not mutually exclusive. Cohorts could report more than one method. Personalised letters were the most common method for individual‐level return, with phone calls mainly used for clarification or follow‐up. All cohorts returning group‐level results used newsletters, often combined with email and in‐person meetings.

**Figure 1:**
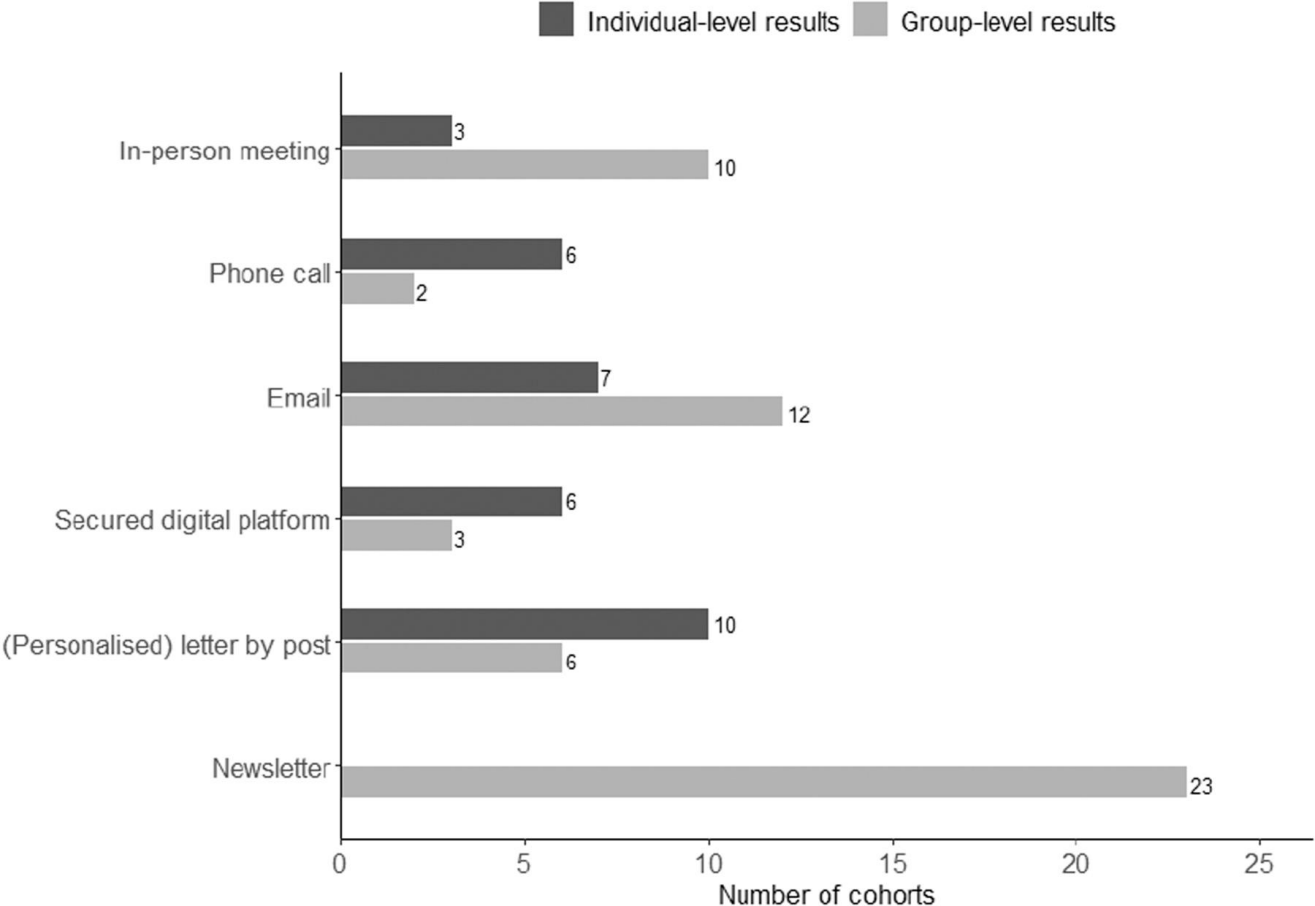
Delivery methods used by cohorts to return individual‐level and group‐level research results.

The organisation of result return differed by result level (Tables 2 and 3). For individual‐level results, particularly laboratory or clinical measurements, a medical doctor was typically involved in reviewing and approving results before they were communicated to participants. For patient‐based cohorts, the return of individual results was not organised as a separate research activity but was integrated into routine clinical care. In population‐based cohorts, research staff often supported the return process, while medical oversight was retained for results considered clinically relevant. For group‐level results, responsibility was more commonly distributed across cohort teams. In many cohorts, principal investigators, study coordinators, and research staff contributed to preparing and disseminating group‐level information. A smaller number of cohorts reported having dedicated communication structures, such as communication or working groups, to support group‐level return. Examples included NMCB, the MS Cohort and NAR, where content development and dissemination were organised collaboratively. In PSA/113, group‐level communication was coordinated in line with the cohort's broader societal mandate, with study leadership and expert committee involved in dissemination through reports, webinars, and other public‐facing channels targeting the broader society as well as the cohort participants.

### 3.4 Reporting of Incidental Findings by Cohorts

Incidental findings are not included in our overview above. All cohorts, apart from OMEGA, reported that unexpected or abnormal findings identified during research assessments are communicated to ensure that participants are made aware. In ADC, individual research results are available to participants through the patient portal as part of routine clinical care. Any unexpected or abnormal findings are evaluated by a medical doctor and, if judged clinically relevant, participants are contacted directly, and the information is shared with their GPs to ensure appropriate follow‐up. Similarly, in ENIGI, data are collected as part of standard clinical care, and all findings are visible to the treating clinician. Where relevant, results are communicated to the participant's GP in accordance with standard care procedures. Due to the retrospective nature of the OMEGA study, incidental findings are not applicable; consequently, no results of this type are returned to participants.

### 3.5 Assessment of Participant Preferences

11 cohorts reported having assessed participants’ preferences regarding the return of research results, either directly through questionnaires or indirectly through participatory activities. Several cohorts used dedicated surveys or questionnaire items to assess preferences, including preferred communication channels and formats for both individual‐ and group‐level results (NL‐SH), preferred delivery methods at the end of data collection interviews (NESDO) and items added to the most recent data collection wave (NESDA). Other cohorts assessed preferences through participant panels or advisory boards (NAR and NMCB). In ACS, preferences are collected periodically through a satisfaction questionnaire and an annual community advisory board meeting. Some cohorts organised co‐creative or participatory activities, such as an anniversary festival where participants were consulted on which results they considered meaningful and how these should be communicated (ABCD), or through ongoing dialogue with affiliated organisations and participating groups (In Kaart and HELIUS). In ADC, communication preferences are assessed within routine clinical practice, although not specifically linked to research result return. In NMCB, patient representatives actively advise on and co‐author communication materials, guided by a communication handbook outlining agreed procedures and style.

### 3.6 Motivations for Returning Research Results

Figure 2 summarises reported motivations. Cohorts could report multiple motivations, and these were assessed at the cohort level without distinguishing between individual and group‐level return. In addition to the predefined response options shown in the figure, several cohorts reported context‐specific motivations, which are described below. The most frequently reported motivation was enhancing participant engagement and retention, reported by 21 cohorts. Many cohorts described return of results as a way to acknowledge participants' contributions and support long‐term involvement. Ethical responsibility to participants was also commonly reported, with 19 cohorts indicating that returning results was considered an ethical obligation of research. Several cohorts elaborated on this motivation. For example, ADC described return of results as reflecting a responsibility for scientists to communicate what has been learned from participants' data, particularly at the group level. A similar rationale was reported by OMEGA, where representatives emphasised the importance of sharing knowledge generated using participant data.

**Figure 2:**
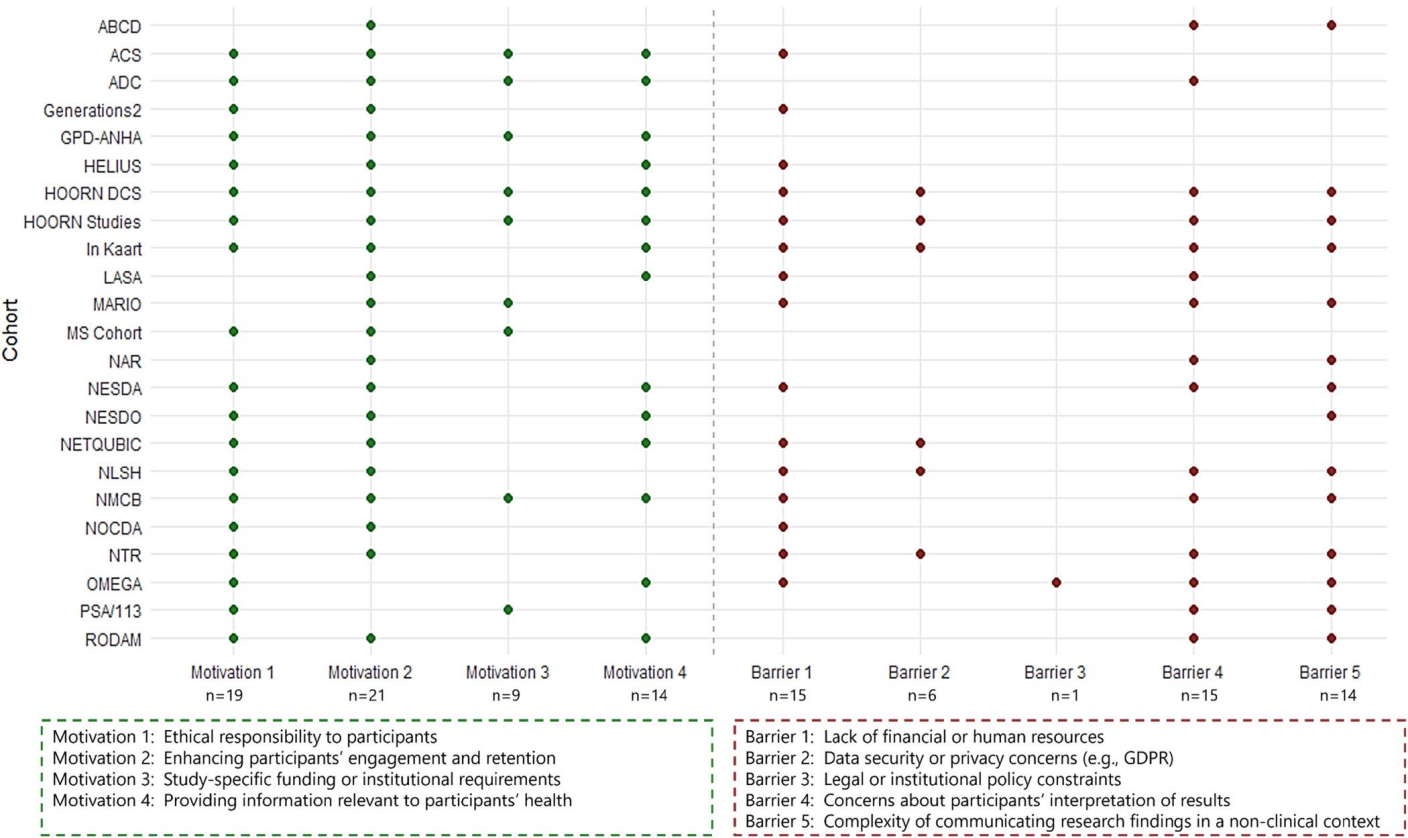
Reported motivations and barriers for return of research results of the 23 cohorts that returned results to participants.

Other cohorts reported informational motivations. For example, in NAR, results are returned to provide participants with insight into overall wellbeing and comparison with peers, without presenting these as clinical feedback. In NESDO, participants were provided with a written summary at the end of the data collection interview, which they are asked to review and confirm for accuracy before the interview is concluded.

### 3.7 Barriers for Returning Research Results

Several barriers were reported across cohorts (Figure 2). Lack of financial or human resources and concerns about participant interpretation were each reported by 15 cohorts. Complexity of communicating research findings in a non‐clinical context was mentioned by 14 cohorts. Cohorts noted that returning results, particularly through newsletters or tailored communication materials, requires sustained staff time for content preparation, review, coordination and delivery. At least one cohort noted that budget cuts raised uncertainty about sustaining current communication with participants.

Concerns about interpretation and communication were particularly evident for cohorts collecting questionnaire‐based, cognitive or behavioural data, where results do not have a straightforward clinical meaning. For instance, LASA, NTR, and NESDA described difficulties in summarising such results without adequate context, noting that isolated scores may be hard for participants to interpret. A related concern is the risk of causing distress or offence when returning scores on traits such as personality or cognitive ability, where certain scores may be perceived negatively. NESDA specifically reported that providing summary scores without clinical explanation could easily lead to misunderstanding, especially given the absence of formal diagnoses. Several cohorts also described ethical tensions related to potential behavioural change following result return. ABCD and the HOORN Studies reported concerns that providing certain results could unintentionally influence participants' behaviour, potentially affecting the observational nature of the study. In Kaart reported similar concerns and noted challenges in balancing relevance across diverse participant groups and affiliated organisations. A related challenge arises in cohorts with ethnically diverse study populations, where communication materials must be adapted across languages and cultural contexts. HELIUS, for example, reported efforts to provide materials in multiple languages to ensure results are accessible to all participants.

Barriers related to topic sensitivity, practical logistics and privacy were also reported, though by fewer cohorts. PSA/113 reported that the sensitivity of suicide‐related research requires specialised expertise, careful wording, and active media coordination to prevent unintended effects such as imitative behaviour. Practical and logistical challenges included delivery failures when GP registrations had changed (NET‐QUBIC), privacy concerns around shared family contact details and secure digital platforms (NTR), multi‐country organisational constraints (ENIGI) and institutional uncertainty about whether communication should be issued on behalf of the study or the host institution (OMEGA).

Several cohorts shared informal observations about participant responses to returned results, although none reported formal evaluation of impact. In HELIUS, receiving results was not a primary driver of participation, although members of the participant panel indicated that receiving results was important for continued involvement. The HOORN Studies reported that participants valued periodic check‐ups. Some cohorts observed that receiving results appeared to support continued participation. In NESDA, some participants explicitly described receipt of laboratory results as a key reason for participation, with staff reporting occasional contact when results were delayed. Similar observations were reported by NET‐QUBIC and NTR. In contrast, participation in the MS Cohort was primarily shaped by health status rather than result return. One cohort noted that results were sometimes shared beyond the individual participant; in PSA/113, family members occasionally shared findings with others in similar situations. Across cohorts, these observations were based on routine interactions rather than systematic assessment, and no cohort reported formally assessing the impact of result return on participation, engagement or retention.

## 4 Discussion

The aim of this study was to provide a descriptive overview of current practices for returning research results to participants in cohort studies, focusing on *what* results are returned, *when* they are communicated, *how* they are delivered, and the motivations and barriers underlying these practices. Across 24 cohorts, result return was widely practised but implemented in diverse ways: group‐level return was near‐universal and relatively consistent, most often through newsletters, while individual‐level return was less frequent, more selective and shaped by cohort design, clinical context and available resources.

### 4.1 What Is Returned

The tendency to restrict individual‐level feedback to easily interpretable measures is consistent with earlier studies showing that participants use laboratory results as informal health checks, often in consultation with their healthcare providers [48]. At the same time, many cohorts refrained from returning questionnaire scores because these results were considered difficult to interpret. This selective approach reflects concerns reported in the literature about the risk of misunderstanding when returning findings without clear clinical meaning or validated reference thresholds [49].

In patient cohorts, individual‐level return was commonly embedded within clinical care, with treating clinicians responsible for communicating results as part of standard diagnostic or follow‐up processes. This aligns with earlier work showing that, in clinical research settings, the boundary between research and care is often blurred, and return of individual findings is shaped by existing clinical infrastructures and professional responsibilities [50]. In such contexts, individual‐level return is less a discrete research activity and more an extension of established care pathways, which may lower practical and ethical barriers to disclosure compared with population‐based cohort studies.

The near‐universal return of group‐level results is consistent with evidence indicating broad support for group‐level return, viewed as less sensitive than individual feedback while fulfilling expectations of transparency and respect for participants' contributions [11, 51, 52]. Studies have shown that participants value receiving information about what has been learned from the research they contributed to, even when this information does not have direct personal health implications [53, 54]. This extends beyond actionable results to include updates on how their data are used [55]. The use of lay language and accessible formats aligns with recommendations for tailoring communication to non‐specialist audiences and avoiding technical or clinical framing when sharing research findings [56]. At the same time, existing studies note that, despite broad normative support for returning group‐level results, practices remain heterogeneous and are often shaped by available resources, communication expertise and institutional priorities [57]. Our findings extend this literature by demonstrating that group‐level return of results is widely implemented within cohort research, with broadly consistent practices but some variation in scope and format.

### 4.2 When Results Are Returned

Our findings suggest that the optimal timing for returning research results remains unclear. While individual‐level results were often returned soon after data collection or embedded within clinical routines, this approach reflects practical constraints rather than evidence‐based guidance on when participants benefit most from receiving results. Delayed return may conflict with participant expectations shaped by clinical care, where results are typically available within days [14, 58, 59]. These tensions are particularly relevant in research settings, where data processing may occur in batches or involve complex analyses, making rapid return unrealistic. At the same time, frequent or detailed result return can place substantial demands on research teams. For group‐level results, timing was shaped more by communication planning than by data availability, which suggests that result return is as much an organisational and communicative task as a scientific one. Together, these findings suggest that timing decisions involve balancing participant expectations, data complexity, and resource constraints, rather than following a single optimal model.

### 4.3 How Results Are Returned

Decisions about who receives research results are shaped by study design, societal role, and regulatory context rather than following a single uniform model. Individual‐level results were primarily returned to participants and, in many cohorts, also GPs, reflecting an effort to situate research feedback within existing care pathways. In cohorts involving children, recipient roles may shift as participants reach age thresholds. Regulatory frameworks such as those set by the Dutch Central Committee on Research Involving Human Subjects can guide these transitions. Nevertheless, they remain administratively complex and labour‐intensive, requiring cohorts to actively track participant ages relative to when results become available. At the group level, results returned followed a broader dissemination logic. While participants remained a central audience, several cohorts shared findings with wider stakeholders, particularly where research had clear societal relevance. PSA/113 provides a prominent example, where group‐level findings are disseminated to inform suicide prevention efforts and directly support policymaking [33, 60]. Similarly, LASA disseminates findings directly to the Dutch Ministry of Health, Welfare and Sport through factsheets developed in co‐creation with the ministry. In these settings, return of results serves not only participant engagement but also broader public health responsibilities.

Returning individual‐level results requires careful handling of sensitive personal information, and cohorts that do so invest substantial effort in secure communication. Potentially identifiable data are encrypted, and access is highly restricted, reflecting deliberate organisational choices to meet privacy requirements and maintain participant trust when returning results [31]. These measures reflect both General Data Protection Regulation requirements and ethical concerns about confidentiality and data security, and they create practical barriers to wider implementation of individual‐level return, especially for cohorts with limited resources.

The organisation of result return reflects different logics for individual‐level and group‐level practices. Involving medical professionals and GPs anchors individual‐level feedback within existing care pathways and mitigates risks of misinterpretation, while dedicated communication teams and participant representatives reflect a more institutionalised approach to group‐level return. Such arrangements may support clear and careful communication, informed by an awareness that research findings can have broader social effects. This was particularly evident in PSA/113, where communication experts helped ensure that dissemination of suicide‐related findings avoided unintended harmful effects such as imitative behaviour [61]. While these approaches may enhance responsible communication, they also require specialised expertise, coordination, and resources that are not available to all cohorts.

### 4.4 Assessment of Participant Preferences

Previous studies show that participants' preferences regarding what results they wish to receive and how they wish to receive them can vary substantially, depending on study context, perceived relevance, and individual characteristics. Incorporating these preferences into communication strategies has been linked to greater perceptions of respect, transparency and engagement, even when assessed indirectly or through representative mechanisms such as advisory panels. The approaches observed in this study show patterns described in the literature, where preference assessment is often embedded within broader participatory activities rather than implemented as standalone procedures [54]. Co‐creation activities, participant panels, and advisory groups are described as practical ways to align result communication with participant expectations, particularly in longitudinal cohort studies [2, 13, 62]. Within NAR, for example, autistic individuals are involved throughout the research cycle, both as members of the research team and through co‐creation panels, advisory groups and ambassador roles. These approaches suggest a shift from one‐way dissemination toward more dialogical models of result return, in which participants contribute to how findings are communicated and contextualised. Overall, while assessing participant preferences does not guarantee increased participation or retention, it can contribute to more acceptable and trusted communication practices, particularly when returning results that may be complex or sensitive [54, 63, 64].

### 4.5 Motivations and Barriers for Returning Results

Across cohorts, motivations for returning research results reflect norms about how research relates to participants and society. Result return signals a shift toward recognising participants as contributors with legitimate expectations of being informed about the use and outcomes of their data, and functions as a mechanism for maintaining trust in long‐term research infrastructures. Ethical responsibility is framed not only as an obligation toward individual participants, but also as a commitment to transparency and accountability in health research [65]. How these ethical motivations are enacted depends on study context. In some cohorts, particularly those addressing socially sensitive or policy‐relevant topics, motivations extend beyond participants to include responsibilities toward communities and decision‐makers. In these settings, result return also requires careful communication strategies to balance transparency with the potential for unintended harm [54]. Beyond ethical considerations, funder requirements further reinforce these expectations. Dutch and European funders require researchers to demonstrate societal impact and to communicate findings beyond the scientific community. Yet meeting these expectations demands specialised expertise, coordination and resources that are not available to all cohorts, a tension that is central to the barriers discussed below.

Barriers to returning research results reflect a structural tension between growing expectations of participant‐centred research and the limited resources available to support it. Returning results requires sustained investments of time, expertise and funding, particularly when communication materials must be tailored, reviewed, and delivered responsibly. Yet this work is rarely recognised as a core research activity by funders or institutions. Instead, responsibility is often placed on individual study teams, who are expected to absorb these efforts alongside data collection, analysis and reporting. In the current Dutch context, where budget cuts in research and higher education further constrain capacity, this expectation becomes increasingly difficult to meet. As a result, some cohorts are able to sustain result return practices due to stable funding or dedicated staff, while others face uncertainty about continuity or are forced to scale back communication efforts. This unevenness suggests that result return is not only an ethical or methodological issue, but also an infrastructural one that requires systemic support rather than reliance on individual goodwill.

Another major barrier concerns the interpretation and communication of research findings that lack clear clinical meaning. These concerns highlight a gap between the complexity of health research and participants' ability to meaningfully interpret such information without guidance [66]. While involving participants more actively through co‐creation or advisory structures is often proposed as a way to improve relevance and clarity, evidence on how to do this effectively remains limited [67]. Preferences for what information is useful, how it should be framed, and through which channels it should be delivered are likely to differ across age groups, educational backgrounds, and cultural contexts [68].

### 4.6 Strengths and Limitations

Our study has several limitations. First, all information was based on self‐report by cohort representatives, which may not fully capture informal practices or variations within teams. At the same time, cohort profiles and published documentation rarely describe result return practices in sufficient detail, making direct interviews with representatives the most feasible approach to obtain this information. Similarly, we did not collect data from participants themselves on how result return practices are experienced or whether they meet participant expectations. A further consideration concerns the generalisability of our findings. Although the 24 cohorts affiliated with ACH are led by principal investigators from different institutions and, in some cases, international consortia, they are part of a shared regional network. Informal overlap in staff and leadership across cohorts within this network may have introduced some degree of shared practice, potentially making result return approaches more similar than would be observed across a broader and more geographically diverse sample of cohort studies. In addition, certain practices may reflect Dutch regulatory requirements, such as those set by the Central Committee on Research Involving Human Subjects, which may differ from requirements in other jurisdictions. These factors should be considered when interpreting the generalisability of our findings to cohort research in other national or institutional contexts. Taken together, these limitations point to the value of complementary qualitative research from both researcher and participant perspectives to better understand how result return is implemented and valued in practice. A key strength of this study is the inclusion of 24 cohorts spanning a broad range of study designs, health domains and study settings, allowing for a diverse descriptive overview of current practices. This overview may inform future research aimed at more detailed examination of specific result return approaches across different cohort contexts.

### 4.7 Future Directions

This overview of current result return practices may serve as a starting point for further development of shared guidance tailored to cohort research. Several concrete directions merit attention. First, the variation in practices observed across cohorts suggests a need for guidance that can support cohorts in making informed decisions about what to return, when and how, while remaining flexible enough to accommodate differences in study design and context. Second, such guidance should be developed with meaningful participant input; co‐creation approaches, already employed by several cohorts in this study, offer a promising avenue for developing communication strategies that reflect diverse needs and preferences without assuming uniform health literacy. Third, dedicated funding mechanisms are needed to support sustainable participant communication, as long as result return is not recognised as a core research activity. Fourth, future research should evaluate the impact of different result return approaches, including portals, letters, newsletters and in‐person meetings, on participant trust, engagement, retention and understanding, and examine how these approaches are experienced by different participant groups. Qualitative research from both researcher and participant perspectives would further deepen this understanding. Addressing these directions may support result return practices that are ethically grounded, practically feasible and responsive to the needs of both participants and research teams [69].

## 5 Conclusion

This study provides an overview of how longitudinal cohort studies approach the return of research results. Result return is widely practised but implemented in diverse ways, shaped by study design, study population, research content, links to clinical care, available resources and perceived ethical responsibilities. Cohorts balance expectations to share results with participants against practical constraints related to time, funding, expertise and communication complexity. Our findings also suggest that results return cannot be treated as a uniform practice. Individual‐level and group‐level return follow different logics and require different forms of organisation, expertise and oversight. While some cohorts have developed structured approaches, including dedicated communication teams or participatory mechanisms, others rely on ad hoc solutions embedded within existing research or clinical workflows.

## Author Contributions

**Yuwei Qi:** conceptualisation, methodology, writing – original draft, writing – review and editing. **Marieke F. van Wier:** conceptualisation, writing – review and editing, resources. **Femke Jansen:** conceptualisation, writing – review and editing, resources. **Marleen van der Horst:** methodology, writing – review and editing, resources. **Charles Agyemang:** writing – review and editing. **Elias Balt:** writing – review and editing. **Marieke M. T. Blom:** writing – review and editing. **Cyrina Brouwer:** writing – review and editing, resources. **Jos A. Bosch:** writing – review and editing, resources. **Anouk den Braber:** writing – review and editing, resources. **Felix P. Chilunga:** writing – review and editing, resources. **Udi Davidovich:** writing – review and editing, resources. **Merijn Eikelenboom:** writing – review and editing. **Awa A. Gassama:** writing – review and editing, resources. **Mirjam I. Geerlings:** writing – review and editing, resources. **Janneke Heijne:** writing – review and editing, resources. **Melany Horsfall:** writing – review and editing, resources. **Martijn Huisman:** writing – review and editing. **Kim M. Jonkman:** writing – review and editing, resources. **Joep Killestein:** writing – review and editing. **Tamira K. Klooker:** writing – review and editing. **Anitra D. M. Koopman:** writing – review and editing, resources. **Sophia E. Kramer:** writing – review and editing. **Jonathan Krikeb:** writing – review and editing, resources. **Franca H. Leeuwis:** writing – review and editing, resources. **Lannie Ligthart:** writing – review and editing, resources. **Saskia Y. M. Mérelle:** resources, writing – review and editing. **Ghiselinde B. G. M. Meijs:** writing – review and editing. **Tom Molenaar:** resources, writing – review and editing. **Mirjam Oosterman:** writing – review and editing. **Patricia van Oppen:** resources, writing – review and editing. **Nadine P. G. Paans:** resources, writing – review and editing. **Brenda W. J. H. Penninx:** writing – review and editing. **Didi Rhebergen:** resources, writing – review and editing. **Femke Rutters:** resources, writing – review and editing. **Yola Sol:** resources, writing – review and editing. **Mandy Spaan:** resources, writing – review and editing. **Eva M. M. Strijbis:** writing – review and editing. **Geertje Marije Takkenberg:** writing – review and editing. **Betty M. Tijms:** resources, writing – review and editing. **Natasja D. Vijfschagt:** resources, writing – review and editing. **Tanja G. M. Vrijkotte:** resources, writing – review and editing. **Marlies J. Visser:** writing – review and editing. **Eco J. C. de Geus:** writing – review and editing, writing – original draft. **Cécile R. L. Boot:** writing – original draft, writing – review and editing. **Emiel O. Hoogendijk:** conceptualisation, methodology, writing – review and editing, writing – original draft.

## Conflicts of Interest

The authors declare no conflicts of interest.

## Data Availability

The data that support the findings of this study are available upon request.

## 1

This questionnaire was administered to representatives of each cohort affiliated with the Amsterdam Cohort Hub (ACH) to document current practices for returning research results to participants. The questionnaire was sent in advance of an individual interview, during which responses were clarified and elaborated where needed.

*Section 1. Return Practices*
For the purposes of this questionnaire, ‘research results’ can include a wide range of information. These can be broken down into two main categories:

I. Individual‐level results: These are results that are specific to a single participant. Examples include:
  ◦ Personalised lab values (e.g., blood pressure, cholesterol levels).
  ◦ Individual genetic findings (e.g., a specific gene variant).
  ◦ Scores from a cognitive test or survey completed by the participant.
  ◦ A participant's own body measurements (e.g., BMI).
II. Aggregate‐level results: These are results that summarise findings for the entire study group, not an individual. Examples include:
  ◦ A newsletter or report detailing the overall progress of the study.
  ◦ A (lay‐language) summary of a published research paper based on the study data.
  ◦ Statistical findings about the entire cohort (e.g., ‘The average BMI for participants in the xxx cohort was 27.5’).

1. Do you return (research) results to participants?
  a. Yes
  b. No (Move to section 3)

2. Do you return results at the individual level?
  a. Yes
    If yes, please specify
  b. No
3. Do you return results at the aggregate/group level?

4. Please specify at what stage(s) results are returned, and if there is a timeline (e.g., within 6 months of data collection, at study closeout, no specific timeline).
5. To whom do you return results?
  a. Participant?
  b. Healthcare provider (e.g. GP)
  c. Family members? (e.g. in the case of studies among children)
  d. Other, please specify
  a. Yes
    If yes, please specify
  b. No

6. What methods do you use to return results? (Check all that apply, and please describe if you select ‘Other’)
  a. Newsletter
  b. Personalised letter by mail to xxx
  c. Secure digital platform/portal
  d. Email
  e. Phone call
  f. In‐person meeting
  g. Other: (Please specify)

7. Have you assessed participant preferences for receiving results (e.g., portal, email, letter)?
  a. Yes
    If yes, how were these preferences measured, and do they vary by participant characteristics (e.g., age, education, digital literacy)
  b. No
8. Who is primarily responsible for the content of returning results (e.g., PI, data manager, study coordinator)?
9. Who is responsible for the organisation/delivery of the return (e.g., communication, scheduling)

Section *2. Participant Inclusion*

1. Are all participants eligible to receive results, or only a subset?
  a. Yes
  b. No
2. Can participants opt out of receiving results?
  a. Yes
  b. No
3. Have you observed any impact of returning results on participant behaviour or study outcomes (e.g., retention, compliance)?

*Section 3. Motivations and Barriers*

1. What are your primary motivations for returning results? (Check all that apply, and please elaborate on your reasoning)
  a. Ethical responsibility to participants
  b. Enhancing participant engagement and retention
  c. Study‐specific funding or institutional requirements
  d. To provide information that may be clinically relevant to participants' health
  e. Other: (Please specify)

2. What are the main barriers you have encountered in returning results? (Check all that apply, and please provide a brief description of the challenge)
  a. Lack of financial or human resources
  b. Data security or privacy concerns (e.g., GDPR)
  c. Legal or institutional policy constraints
  d. Concerns about clinical interpretation and potential for participant anxiety
  e. Complexity of communicating research findings in a non‐clinical context
  f. Other: (Please specify)

3. Do you have a process to collect feedback from participants on the results they receive?

Open question: Do you feel this questionnaire has missed any important questions or topics related to returning research results? If so, please feel free to share your suggestions and comments.
